# Probing the heterogeneous structure of eumelanin using ultrafast vibrational fingerprinting

**DOI:** 10.1038/s41467-020-18393-w

**Published:** 2020-09-11

**Authors:** Christopher Grieco, Forrest R. Kohl, Alex T. Hanes, Bern Kohler

**Affiliations:** Department of Chemistry and Biochemistry, The Ohio State University, 100 West 18th Avenue, Columbus, Ohio 43210 USA

**Keywords:** Excited states, Infrared spectroscopy

## Abstract

Eumelanin is a brown-black biological pigment with sunscreen and radical scavenging functions important to numerous organisms. Eumelanin is also a promising redox-active material for energy conversion and storage, but the chemical structures present in this heterogeneous pigment remain unknown, limiting understanding of the properties of its light-responsive subunits. Here, we introduce an ultrafast vibrational fingerprinting approach for probing the structure and interactions of chromophores in heterogeneous materials like eumelanin. Specifically, transient vibrational spectra in the double-bond stretching region are recorded for subsets of electronic chromophores photoselected by an ultrafast excitation pulse tuned through the UV-visible spectrum. All subsets show a common vibrational fingerprint, indicating that the diverse electronic absorbers in eumelanin, regardless of transition energy, contain the same distribution of IR-active functional groups. Aggregation of chromophores diverse in oxidation state is the key structural property underlying the universal, ultrafast deactivation behavior of eumelanin in response to photoexcitation with any wavelength.

## Abstract (web summary)

Eumelanin protects cells from sun damage and is promising for energy conversion applications, but its structure and excited state dynamics are elusive. Here the authors shed light on both aspects combining selective excitation of UV- and visible-absorbing chromophores with time-resolved infrared spectroscopy.

## Introduction

Melanins are multifunctional, biological pigments found in virtually every organism. Melanin pigments protect cells from damage by sunlight and by oxidants, but melanins are also associated with major human diseases. The brown–black pigment known as eumelanin plays an important, but uncertain role in melanoma^1,2^, whereas the chemically similar neuromelanin is present at greatly reduced levels in the brains of patients with Parkinson’s disease^3^. Very recently, eumelanin-based materials have attracted widespread interest for solar energy conversion, electrochemical energy storage, and biomedical applications^4–6^. Because it absorbs broadly from the UV to near-infrared spectrum and because of its ability to conduct^7,8^ and store charge, eumelanin is receiving considerable attention for applications including photocatalysis, battery electrodes, and supercapacitors^5^.

**Fig. 1:**
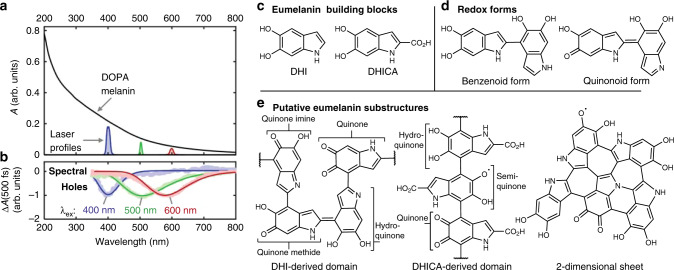
Transient spectral hole burning of DOPA melanin (eumelanin) and putative chromophore structures. **a** Absorption spectrum (black curve) of an aqueous solution of DOPA melanin. Profiles of excitation laser pulses (colored curves) used in femtosecond transient absorption measurements showing their spectral width (arbitrary vertical scaling). **b** Transient spectral holes extracted from transient absorption spectra measured 500 fs after excitation at 400, 500, or 600 nm. **c** Chemical structures of eumelanin building blocks. **d** Dimers of the building blocks, showing two major types of redox forms. **e** A few substructures proposed for DOPA melanin and other eumelanins. Labels indicate redox forms, which vary from least to most oxidized in the order hydroquinone<semiquinone<quinone imine/methide<quinone.

Despite several decades of study, the chemical structure of eumelanin remains elusive and the nature and assembly motifs of its light-absorbing units, or chromophores, are unknown. Understanding of the fundamental chromophores and their interactions is needed to explain eumelanin’s broad and featureless absorption spectrum (Fig. 1a) and its excited state dynamics. This knowledge gap not only impedes understanding of the role eumelanin plays in human disease, but also hinders the rational design of functional materials for energy and biomedical applications^9^.

The chemical disorder model proposed by Tran et al.^10^ postulates that the broad and featureless absorption spectrum of eumelanin is a superposition of absorption bands owing to chemically heterogeneous subunits. Transient electronic spectral hole burning observed in femtosecond laser experiments on eumelanins^11,12^ (Fig. 1b) indicates the presence of distinct chromophores that differ in their absorption spectra. Although these results confirm that eumelanin is a heterogeneous ensemble of chromophores with different lowest-energy electronic transitions, the microscopic properties that differentiate the chromophores and determine their excited state behaviors remain highly uncertain.

Eumelanin is thought to be built from the indolic compounds, 5,6-dihydroxyindole (DHI) and 5,6-dihydroxyindole-2-carboxylic acid (DHICA) (Fig. 1c). Covalently linking these building blocks into protomolecules^13^ tunes their conjugation lengths but preserves their heteroatom-based functional groups (Fig. 1d–e). Importantly, electronic conjugation can extend across multiple indolic groups, depending on redox state (Fig. 1d). The protomolecules likely include a variety of redox-mutable subunits such as hydroquinone, quinone methide, quinone imine, and quinone (Fig. 1e). Noncovalent interactions are thought to further modulate the structure of eumelanin, and a currently favored paradigm is that planar eumelanin protomolecules assemble into nanoaggregates through π–π interactions^13–15^. These aggregates are likely to be heterogeneous not only in terms of their packing structure, but also because they may contain protomolecules that differ in chemical structure and/or redox state.

Investigators have augmented the chemical disorder model by pointing out that electronic conjugation length^16^, redox state variations^16–20^, and excitonic delocalization^14,15^ could all affect the spectral and dynamical properties of eumelanin chromophores, but to an unknown degree. Although it is currently difficult to disentangle these effects, the observation of spectral hole burning^11^ in electronic transient absorption experiments (e.g., Fig. 1b) suggests that different subsets of chromophores in eumelanin can be interrogated spectroscopically. Coupling transient spectral hole burning to a structurally sensitive measurement, such as vibrational spectroscopy, would allow electronic transition energies to be correlated with IR-active functional groups like carbonyls, which are expected to be present in higher amounts in more oxidized protomolecules.

Here, we combine the recently demonstrated ability to selectively excite eumelanin chromophores based on their transition energies^11^ with time-resolved vibrational spectroscopy, a technique that has not been used previously to study any form of melanin. By tuning an excitation pulse from the UV through the visible, subsets of electronic chromophores are selected, and a mid-IR probe pulse detects transient changes in vibrational modes in an approach we term ultrafast vibrational fingerprinting (UVF). This technique, which provides much of the information content of a two-dimensional electronic-vibrational spectroscopy (2D EV) experiment, is used to investigate whether chromophores that absorb at different wavelengths differ in the number and kinds of IR-active functional groups by probing their double-bond stretching modes. Each subset of selected chromophores has the same transient vibrational spectrum, or fingerprint, in the measurement window spanning 1300–1900 cm^−1^ and identical decay kinetics. This suggests that each subset of electronic chromophores contains the same distribution of functional groups as any other set, a property that we propose underlies the universal photoresponse observed in eumelanin^11^.

## Results

### Demonstrating UVF

**Fig. 2:**
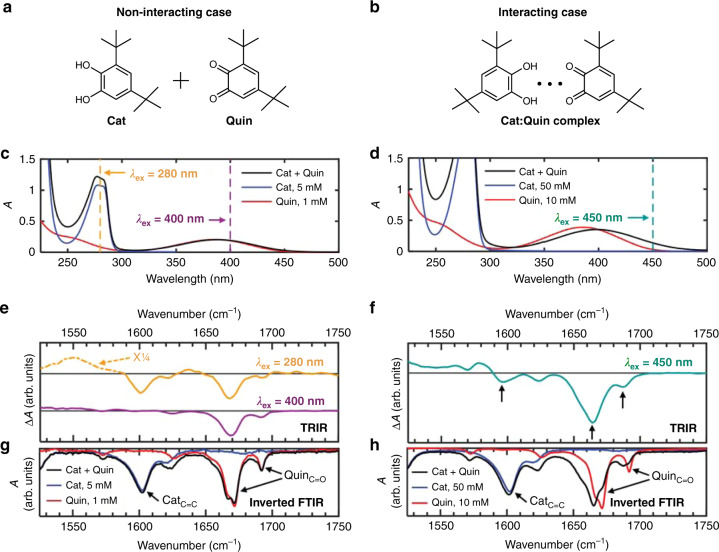
Ultrafast vibrational fingerprinting demonstrated for a model system. Solutions of catechol (Cat) and quinone (Quin) mixtures in cyclohexane contain either **a** non-interacting, isolated molecules, or **b** molecules interacting through intermolecular hydrogen bonds. The Cat:Quin mole ratios were fixed at 5:1. **a**, **b** Chemical structures of Cat and Quin. **c**, **d** UV-vis spectra of cyclohexane solutions of Cat, Quin, and their mixtures, highlighting the excitation wavelengths used in time-resolved infrared (TRIR) spectroscopy experiments. The cell pathlengths used in **c** and **d** were 1 mm and 200 μm, respectively. **e**, **f** TRIR spectra of the mixture solutions averaged from 1–2 ps, recorded at the indicated excitation wavelengths. **g**, **h** Inverted FTIR spectra of cyclohexane solutions of Cat, Quin, and their mixtures.

UVF (see “Discussion” of this method below) was used to correlate electronic and vibrational degrees of freedom in cyclohexane (solvent) mixtures of the catechol (Cat) and quinone (Quin) derivatives with the structures shown in Fig. 2a. These molecules model the dihydroxy and quinone functional groups present in eumelanin^21^. Cat and Quin self-associate to yield hydrogen-bonded heterodimers (Fig. 2b) when their concentrations in cyclohexane are sufficiently high^21^. Here, we study both a dilute (Fig. 2a, c, e, g) and a concentrated (Fig. 2b, d, f, h) mixture.

UV-visible (UV-Vis) absorption spectra (Fig. 2c, d) and FTIR spectra (Fig. 2g, h) were recorded of Cat+Quin mixtures and Cat-only and Quin-only control samples. The UV-vis spectrum of the dilute mixture (Fig. 2c) approximately equals the sum of the spectra of the Cat-only and Quin-only solutions, which have lowest energy bands at 280 nm and 387 nm, respectively. For the spectrum of the concentrated mixture (Fig. 2d), the 387 nm band due to Quin broadens and redshifts to 400 nm, which results from hydrogen bonding between Cat and Quin molecules^21^.

The FTIR spectra of the Cat-only and Quin-only solutions are essentially independent of concentration (Fig. 2g, h). The Cat-only solutions feature a prominent vibrational band at 1603 cm^−1^ (aromatic C=C stretching), whereas the Quin-only solutions feature major bands at 1672 cm^−1^ and 1692 cm^−1^ (C=O stretching). In the dilute mixture, there are minimal changes in the peak positions and lineshapes of the C=C and C=O bands (Fig. 2g). In the concentrated mixture, the C=O bands of Quin downshift to 1665 cm^−1^ and 1688 cm^−1^ and change significantly in lineshape (Fig. 2h) owing to hydrogen bonding between Cat and Quin molecules. Each reported vibrational frequency corresponds to the frequency of maximum intensity for the relevant band.

Figure 2e, f show the time-resolved infrared (TRIR) spectra of the Cat+Quin mixtures averaged over 1–2 ps. The dilute mixture was excited either at 280 nm, where Cat and Quin molecules absorb, or at 400 nm, where only Quin molecules absorb (dashed vertical lines in Fig. 2c). Exciting at 280 nm produces negative peaks in the transient absorption (Δ*A*) spectrum at 1600 cm^−1^, 1668 cm^−1^, and 1693 cm^−1^ (orange trace in Fig. 2e), which are close in frequency to ones measured in the FTIR spectra of Cat, Quin, and their dilute mixture (Fig. 2g). Exciting at 400 nm produces negative peaks at 1668 cm^−1^ and 1693 cm^−1^, which are close in frequency to peaks in the Quin FTIR spectrum (red trace in Fig. 2g), but not at 1600 cm^−1^, the frequency of the band assigned to Cat. The TRIR spectrum of the concentrated mixture excited at 450 nm (dashed vertical line in Fig. 2d) has negative peaks at 1664 cm^−1^ and 1688 cm^−1^, and, most significantly, at 1597 cm^−1^ (see arrows in Fig. 2f). These peaks closely match ones of Cat and Quin in the FTIR spectrum of the concentrated Cat+Quin mixture (black trace in Fig. 2h).

### Electronic-vibrational correlations in DOPA melanin

**Fig. 3:**
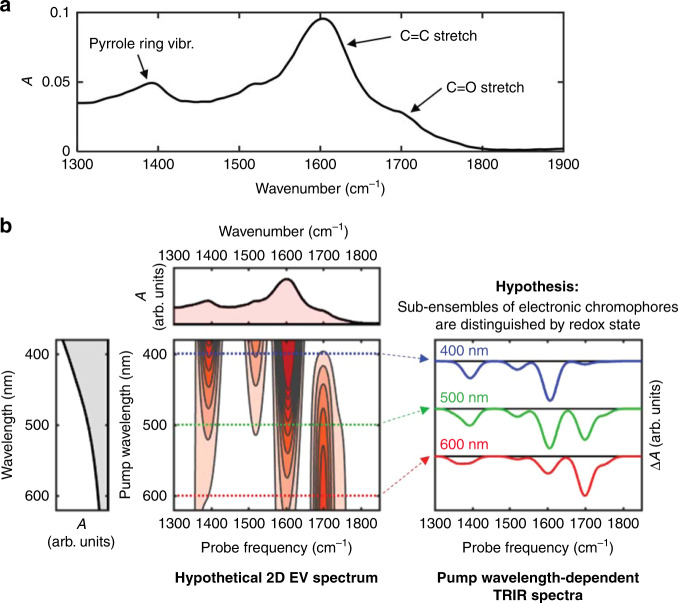
Vibrational spectroscopy of DOPA melanin and UVF predictions. **a** FTIR spectrum of DOPA melanin dissolved in phosphate-buffered D_2_O, including vibrational assignments. **b** Ultrafast vibrational fingerprinting (UVF) spectroscopy vs. two-dimensional electronic-vibrational (2D EV) spectroscopy for a hypothetical sample containing multiple electronic chromophores that can be distinguished by the redox state of their oxidizable IR-active functional groups. With time-resolved infrared (TRIR) spectroscopy (right), a subensemble of eumelanin chromophores is photoselected using a particular visible wavelength and its time-resolved vibrational spectrum is measured. These spectra are essentially horizontal slices through the hypothetical 2D EV spectrum (left). The 2D signals are assumed to only contain negative absorptive (bleaching) contributions for simplicity. Note that the presence of positive signal contributions from new vibrational frequencies in excited electronic states can alter the positions of the negative bands.

The FTIR spectrum of the synthetic eumelanin, DOPA melanin (Fig. 3a), shows several bands or shoulders in the 1300–1900 cm^−1^ region. The most prominent features at 1391 cm^−1^, 1604 cm^−1^, and 1706 cm^−1^ are labeled in Fig. 3a by mode assignments for natural Sepia melanin reviewed by Centeno and Shamir^22^. Fig. 3b depicts hypothetical correlations between the visible absorption spectra and the vibrational spectra of DOPA melanin, based on the hypothesis that electronic chromophores are differentiated by IR-active functional groups. Importantly, the vibrational frequencies of double-bond stretching modes such as the C=O, C=N, C=C, and ring stretching modes probed here are very sensitive to the oxidation state of the parent compound as illustrated by calculations for the various redox forms of DHI^23^. If chromophores that absorb at longer wavelengths in the electronic absorption spectrum are more oxidized, then bleaching of the relatively high-frequency C=O stretching modes of their quinone groups should be prominent (red trace in Fig. 3b), whereas if blue-absorbing electronic chromophores are more reduced, then minimal or no C=O stretching might be detected (blue trace in Fig. 3b).

**Fig. 4:**
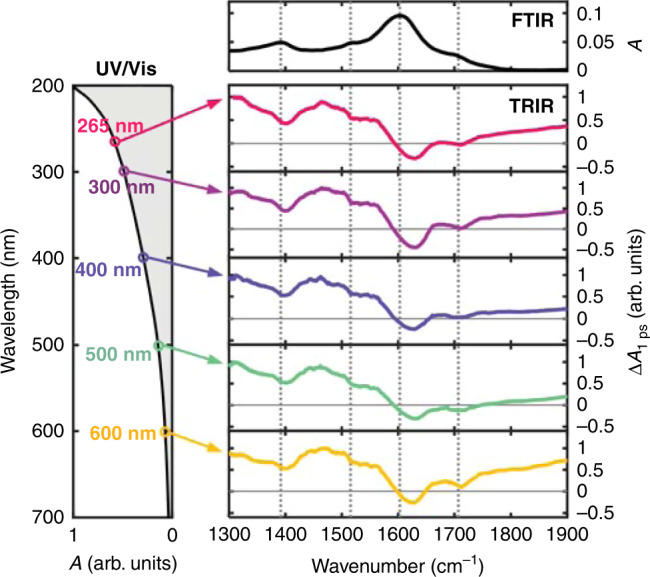
Ultrafast vibrational fingerprinting of DOPA melanin. Time-resolved vibrational spectra (TRIR) are shown at 1 ps after photoexcitation at the wavelengths indicated on the left. Spectra are normalized for clarity. Vertical dotted lines indicate ground state vibrational peak positions. The electronic (UV-vis) and vibrational (FTIR) absorption spectra of DOPA melanin are shown on the left and top, respectively, with axes that are linear in absorbance.

Normalized TRIR spectra of a DOPA melanin solution recorded 1 ps after photoexcitation at 265 nm, 300 nm, 400 nm, 500 nm, or 600 nm are identical within experimental uncertainty (Fig. 4, also see Supplementary Note 1). Each TRIR spectrum in Fig. 4 nearly mirrors the FTIR spectrum and exhibits negative-going peaks located at similar frequencies (see the dashed vertical lines in Fig. 4). The TRIR peak frequencies do not vary as the excitation wavelength is changed.

### Excited state relaxation kinetics in DOPA melanin

**Fig. 5:**
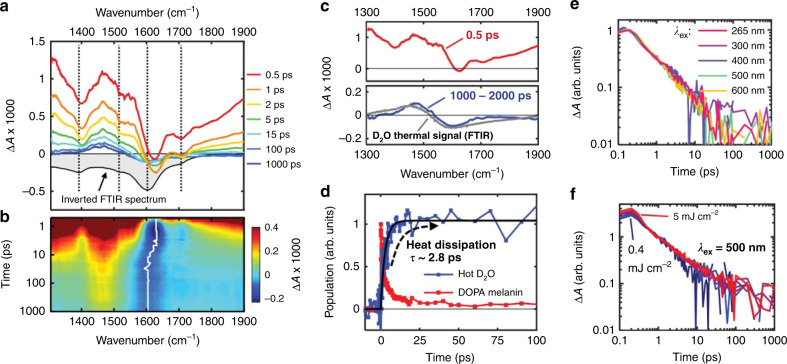
Time-resolved infrared (TRIR) spectra and kinetics of DOPA melanin. **a** Transient absorption spectra of a solution of DOPA melanin dissolved in phosphate-buffered D_2_O, shown at several time delays after 265 nm excitation. Vertical dotted lines indicate ground state absorption peak positions. **b** The corresponding two-dimensional transient absorption map, showing Δ*A* × 1000 with a colormap from −0.25 (blue) to 0.4 (red). The white trace shows the kinetics of the minimum signal intensity of the transient absorption spectrum. **c** Basis spectra selected for spectral modeling. The gray spectrum shows the D_2_O thermal signal estimated using temperature-dependent FTIR spectra (see Supplementary Fig. 4). **d** Isolated kinetics of the DOPA melanin signal compared with the D_2_O thermal signal. The black trace is a fit to the latter with an exponential growth model, and the corresponding time constant (*τ*) is shown. **e** Normalized excitation wavelength-dependent transient absorption kinetics of DOPA melanin probed at 1310 cm^−1^. **f** Power-dependent kinetics probed at 1310 cm^−1^ and recorded using 500 nm excitation, normalized at 1 ps. Seven traces are shown with pump pulse fluences that vary between 0.4 and 5.0 mJ cm^−2^.

Exciting DOPA melanin at 265 nm produces the TRIR spectra in Fig. 5a. All other tested excitation wavelengths produce similar spectral behavior (Supplementary Note 2). The negative-going bands are already present at 0.5 ps and then decay within 15 ps. A concurrent decay is seen for overlapping broad, positive Δ*A* signals spanning the entire spectral window. After 15 ps, the spectra no longer resemble the inverted FTIR spectrum of DOPA melanin, but instead agree with the differential absorption spectrum of heated D_2_O relative to its room temperature spectrum (Fig. 5c and Supplementary Note 3). The evolution of the vibrational feature in the TRIR spectrum near 1600 cm^−1^ is more clearly seen in the two-dimensional map in Fig. 5b. The white line in this map shows the evolution of the negative peak, which shifts from 1627 cm^−1^ (DOPA melanin signal) to 1602 cm^−1^ (hot D_2_O signal).

As explained in detail in Supplementary Note 4, the TRIR spectra can be modeled by contributions from DOPA melanin excited states, D_2_O molecules that are heated by accepting vibrational energy following excited state decay by DOPA melanin, and a spectrally broad background signal (see Supplementary Note 5). Importantly, the kinetics of both solvent heating and excited state decay can be extracted from the data without the need for a kinetic model by least-squares fitting to the basis spectra shown by the red and blue curves in Fig. 5c. The resulting kinetics obtained from global fitting are shown in Fig. 5d. The hot D_2_O signal grows exponentially with a time constant of 2.8 ± 0.4 ps (95% confidence interval estimated from the fit shown by the solid black curve in Fig. 5d).

The normalized TRIR kinetics of DOPA melanin probed at 1310 cm^−1^ are the same for all excitation wavelengths used in this study (Fig. 5e). The kinetics are also independent of excitation fluence when normalized at 1 ps (Fig. 5f). Note that the TRIR decay kinetics probed at 1310 cm^−1^ are devoid of signals from hot D_2_O because there is an isosbestic point in the temperature-dependent D_2_O FTIR spectrum at this frequency (see Supplementary Note 3).

## Discussion

TRIR spectroscopy with UV-vis excitation and mid-infrared probing is used in this study to fingerprint the chromophores in DOPA melanin by monitoring IR-active vibrations that change in response to photoexcitation. For aromatic molecules, photoexcitation typically produces ππ* and nπ* excited states that are delocalized over the full nuclear framework. This causes bleaching of virtually all double-bond vibrational bands in the electronic ground state owing to frequency shifts upon excitation^24^. In a heterogeneous sample like eumelanin, subsets of absorbers can be selectively photoexcited by tuning the excitation wavelength^11^. In the language of hole burning spectroscopy, photoexcitation excites an isochromat of molecules that have a common ability to absorb the pump wavelength^25^. Detecting the IR frequencies bleached by the excitation pulse can provide structural insights through information about the types of functional groups present in the isochromat. We refer to the use of pump wavelength-dependent TRIR spectroscopy for identifying the vibrational signatures of diverse chromophores selected by their transition energies as UVF.

UVF provides some of the same information correlating electronic and vibrational degrees of freedom as two-dimensional electronic-vibrational (2D EV) spectroscopy^26–28^, but is easier to implement. In the frequency-domain realization of 2D spectroscopy, also known as the hole burning method^29,30^, a pump pulse with a bandwidth less than the homogeneous linewidth of an electronic transition of interest excites the sample and burns a hole in its spectrum, and a transient or pump-probe spectrum is recorded. Repeating this procedure at many excitation wavelengths produces a two-dimensional spectrum. Our pump wavelength-dependent TRIR measurements constitute a frequency-domain implementation of 2D EV spectroscopy in which the TRIR spectra are slices^31^ through the full 2D EV spectrum (see Fig. 3b).

Tuning the excitation wavelength selects distinct subsets of absorbers^11^, yet the vibrational fingerprints recorded for isochromats excited from 265 nm to 600 nm are indistinguishable within experimental uncertainty (Fig. 4). This observation disproves the hypothesis that eumelanin chromophores absorbing at different wavelengths can be differentiated based on their oxidizable, IR-active functional groups. Possible explanations for how a constant vibrational response can result, even as the electronic transition energy is tuned, differ depending on the degree of interaction among the chromophores as we discuss next.

Non-interacting electronic absorbers that differ in their vibrational spectra would yield distinct fingerprints as seen in the dilute Cat+Quin mixture. In this case, a 400 nm excitation pulse excites only the oxidized chromophore, Quin, bleaching its C=O bands, but not the C=C bands of Cat (Fig. 2e). Thus, in the absence of interactions, the observation of identical fingerprints in DOPA melanin would indicate that each isochromat, regardless of electronic transition energy, contains the same distribution of IR-active functional groups. However, it is difficult to imagine how chromophores that differ in size, as suggested by wavelength-dependent Raman scattering results^11^, could all have a common IR absorption spectrum. Furthermore, if the chromophores were to remain constant in size, then changes in functional groups would be needed to tune the electronic transition energy, but this would lead to unique IR signatures for different isochromats contrary to the observations.

We propose that coupling among nearby protomolecules in eumelanin provides a natural way to understand how the members of an isochromat collectively give rise to a common vibrational spectrum. TEM^13^ and transient absorption^14^ measurements support the model that eumelanin is made of π-stacked nanoaggregates^32^. Closely spaced chromophores can couple electronically causing new transition energies that differ from those of the separated chromophores. We propose that photoexcitation of multiple coupled eumelanin chromophores produces Frenkel and/or charge transfer (CT) excitons that bleach vibrations associated with each chromophore, similar to how electronically exciting π-stacked nucleobase dimers bleaches the vibrational fundamentals of both bases^24^. Bleaching of the vibrational bands of multiple interacting molecules is also illustrated by the TRIR spectrum of the concentrated Cat+Quin mixture, where preferential excitation of Quin in Cat:Quin complexes leads to bleaching of vibrational modes of both Cat and Quin molecules (Fig. 2f)^21^.

Chromophore size, electronic coupling, and the presence of redox-active functional groups like quinones are all likely to tune the transition energy of a chromophore aggregate. Disentangling their relative contributions is difficult, but because every isochromat contains all possible functional groups, functional group content (quinone, semiquinone, hydroquinone, etc.) is likely less important than size for determining electronic transition energies. Tuna et al. showed in a computational study that increasing the size of a DHI oligomer from 1 to 5 monomer units shifts its lowest transition energy by ~1 eV^16^. Such a shift could tune the energies of eumelanin chromophores through much of the visible spectrum (the energy difference between 400 and 600 nm is ~1 eV).

This conclusion is further supported by wavelength-dependent resonance Raman spectra of eumelanins^33^, including DOPA melanin^11^, which point to the presence of multiple types of chromophores that differ in electronic conjugation length, or sp^2^ domain size^11^. It is important to note that by size we are referring to the conjugation length, which could differ from the physical size of a protomolecule. Instead of tuning transition energies, functional groups could carve out sp^2^ domains of different size as they do in graphene oxide^34^. Alternatively, in aggregates of diverse chromophores, the random presence of several possible functional groups that are equally likely to be reduced or oxidized could cause shifts in transition energies that average to near zero, allowing chromophore size to be the primary factor behind the variation in electronic transition energy.

The time-evolution of the TRIR spectra provide additional insights into the ultrafast relaxation of excited eumelanin chromophores. The TRIR spectral dynamics and kinetics in Fig. 5 reveal that the majority of the population of electronic ground states recovers on the early picosecond timescale, consistent with electronic bleach recovery kinetics^11,35^. TRIR spectroscopy probes thermal dissipation directly by monitoring the growth in the thermal signal of D_2_O, which is complete by ~20 ps. Note that the roughly simultaneous rise of the thermal signal and the decay of the DOPA melanin signal (Fig. 5d) indicate that heat is transferred to the solvent immediately following relaxation of the excited state population. Previous measurements of ultralow photoluminescence quantum yields in eumelanin established the dominance of nonradiative deactivation pathways^36^, which were shown to be ultrafast in pioneering measurements by Simon and co-workers^37^. Although photoacoustic calorimetry experiments were used to infer that these pathways transfer heat to the solvent on a sub-nanosecond timescale^38^, our measurements unambiguously show that heat is rapidly transferred from the DOPA melanin to the solvent via vibrational energy transfer in several picoseconds.

The TRIR spectra mirror much of the structure seen in the inverted FTIR spectrum as expected for bleaching signals, but the Δ*A* signals are positive everywhere except between 1600 and 1650 cm^−1^ (Fig. 4). To maintain the near mirror-image symmetry with the inverted FTIR spectrum, a positive signal contribution that varies slowly with probe frequency must be present. The positive TRIR signals seen above 1750 cm^−1^ occur at frequencies where there is little to no absorption by DOPA melanin in its ground electronic state (see the FTIR spectrum in Fig. 4). We propose that the signal here, which is broader than for typical excited state vibrations, is electronic in origin. The average signal decay measured between 1750–1900 cm^−1^ exhibits stretched exponential kinetics that at every excitation wavelength match the decay kinetics seen at near-infrared probe wavelengths (see Supplementary Fig. 3)^11^. The kinetics at 1310 cm^−1^ (Fig. 5e), which are free of the D_2_O thermal signal (Supplementary Fig. 4), track with the fast decay component of the near-infrared kinetics, except at times <0.5 ps (Supplementary Fig. 3). The matching kinetic behavior indicates that the electronic state responsible for the mid-IR signals is the same one detected in the near-infrared^11^.

The identical TRIR kinetic traces in Fig. 5e indicate that regardless of which set of DOPA melanin chromophores is photoexcited, excited state relaxation proceeds at the same rate. Because every isochromat contains all possible IR-active functional groups, we propose that CT between reduced and oxidized indolic units within chromophores^20^ or between chromophores in aggregates creates CT excitons or polaron pairs (PPs) that decay with common deactivation kinetics^11^. The observation here of an electronic absorption signal in the mid-IR that matches the near-IR transient absorption kinetics we reported recently^11^ supports this assignment. Many computational studies to date consider stacks of chromophores with a fixed redox state^15,16^, and future calculations of coupled chromophores with mixed redox states are needed.

Photoinduced electronic absorption in the mid-IR by polarons and PPs formed via CT is a hallmark of semiconducting polymers^39^. Ultrafast (sub-picosecond) generation of geminate PPs in polymers^40,41^, such as poly(3-hexylthiophene) (P3HT) (<20 fs)^42^, is reminiscent of our findings in DOPA melanin. Geminate PPs show excitation power-independent decay kinetics^39,41,43^ because they decay unimolecularly^41,44^. These observations align well with the excitation power-independent TRIR kinetics at 1310 cm^−1^ of DOPA melanin (Fig. 5f). Furthermore, contacts between reduced and oxidized structures in DOPA melanin can facilitate the CT needed for polaron formation. For example, photoexciting either molecule in the Cat:Quin heterodimer induces an ultrafast comproportionation reaction yielding a semiquinone radical pair state^21^.

Ultrafast recombination of PPs in eumelanin has not been proposed previously and can explain the electronic deactivation responsible for photoprotection. PPs that fail to recombine could lead to the long-lived radical species, such as those detected by electron spin resonance in photoexcited natural and synthetic eumelanins^45^. This proposal differs from the previous assignment of ultrafast nonradiative decay in non-aggregated DHICA oligomers to singlet excited state deactivation^46^. Aggregation of eumelanin subunits enables the ultrafast CT needed to form PPs. Altogether, the TRIR results suggest that the photogenerated species in eumelanin differ strongly from those in non-aggregated model systems, suggesting the importance of considering alternative ultrafast deactivation mechanisms to explain eumelanin photoprotection.

In conclusion, the present UVF results unexpectedly reveal that excited groups of chromophores in a synthetic eumelanin selected by their transition energies have a common TRIR spectrum and decay kinetics. The insight that all chromophore subsets contain the same distribution of functional groups is explained by a model in which chromophores decorated at random with all possible IR-active functional groups are electronically coupled together in aggregates, leading to wavelength-independent excited state relaxation. TRIR spectroscopy directly detects ultrafast thermal dissipation from the relaxed eumelanin chromophores to the solvent and reveals that the photoexcited states in eumelanin may have different electronic character than in monomers and oligomers. Finally, the UVF method can be applied effectively to the study of other heterogeneous carbonaceous systems such as graphene oxide^47^, carbon nanodots^48^, chromophoric dissolved organic matter^49^, and pitch^50^, all of which feature elusive combinations of chromophores and eumelanin-like absorption.

## Methods

### Materials

DOPA melanin was synthesized from L-DOPA using base-initiated oxidative polymerization^11^. First, 1 g of L-DOPA (Sigma Aldrich, ≥98%) was mixed with 200 mL of nanopure water. Then ammonium hydroxide solution (28–30% by mass, Fisher Chemical) was added to the mixture dropwise until the pH was stabilized at 9.5. At this time, the L-DOPA was fully dissolved. The mixture was gently stirred, and air was bubbled for 3 days. Afterward, approximately 200 mL of acetonitrile was added to the reaction mixture, causing the DOPA melanin product to flocculate. The reaction mixture was then centrifuged at 10,000 × *g*, yielding a pellet of the DOPA melanin. After removing the supernatant, the pellet was resuspended in fresh acetonitrile. Centrifugation and resuspension were repeated 2–3 times until the supernatant was clear and colorless and the UV-vis spectrum of the supernatant lacked absorption bands above 250 nm. Finally, the pellet was dried under nitrogen, yielding dark and shiny flakes with high water solubility.

For FTIR and TRIR measurements, DOPA melanin powder was dissolved at 5 mg mL^−1^ in phosphate-buffered D_2_O solution, containing 25 mm each of sodium phosphate monobasic and sodium phosphate dibasic. The pD value of the buffer and DOPA melanin solution was 7.6 ± 0.1, which is close to what is expected in the stratum spinosum and stratum basale in human skin, where melanocytes produce melanins^51^.

The Cat and Quin derivatives, 3,5-di-*t*-butylcatechol and 3,5-di-*t*-butyl-*o*-quinone, respectively, were purchased from Sigma Aldrich (98% purity). Cyclohexane was from Acros Organics (HPLC grade, 99.8%). The dilute Cat and Quin mixture contained Cat and Quin concentrations of 5 mm and 1 mm, respectively. These concentrations were increased tenfold in the concentrated Cat and Quin mixture. Control samples were studied for each mixture, in which each component (Cat or Quin) was present by itself in cyclohexane solution at the same concentration as in each mixture.

### Steady-state spectroscopy

UV-vis absorption spectra were measured using a Cary UV/Vis/NIR spectrometer (Agilent; Santa Clara, CA) set to a 2 nm effective spectral bandwidth. Solutions were measured either in quartz cuvettes or a homemade demountable liquid cell consisting of Teflon spacers and CaF_2_ windows. Pathlengths were selected to ensure that the maximum absorbance did not exceed 1.

IR absorption spectra were recorded using an FTIR spectrometer (FT/IR-4200, JASCO; Easton, MD). The spectral resolution was set to 4 cm^−1^ for DOPA melanin and 0.5 cm^−1^ for the Cat and Quin solutions. Solutions were measured in the same demountable liquid cell described above using a 100 μm spacer for D_2_O solutions and 200 μm or 500 μm spacers for cyclohexane solutions. For DOPA melanin measurements, special care was taken to avoid spurious signals arising from unequal concentrations of HOD and buffer between blank and sample scans.

### TRIR spectroscopy

TRIR spectroscopy was performed using a commercial transient 2D IR spectrometer (2DQuick IR with the 2DQuick Transient add-on module, PhaseTech Spectroscopy; Madison, WI). The ultrafast UV-vis and mid-IR pulses were generated using optical parametric amplifiers (OPAs). Specifically, the output of a Ti:Sapphire laser amplifier (Astrella, Coherent; Santa Clara, CA) was split and sent into an OPA (TOPAS Prime, Coherent; Santa Clara, CA) to generate UV or visible excitation pulses and another OPA with a difference frequency generation (DFG) attachment (TOPAS Prime/DFG, Coherent; Santa Clara, CA) to generate mid-infrared probe pulses. For each excitation wavelength used (265, 300, 400, 500, and 600 nm), the on-axis excitation fluence was between 0.5 and 1 mJ cm^−2^, assuming a Gaussian beam. For power-dependent measurements performed with 500 nm excitation, the on-axis fluence was in the range of 0.4–5 mJ cm^−2^. The magic angle pump-probe condition was used in all cases. The sample pathlength was 100 μm and the samples were continuously recirculated during all scans. We monitored the absorption spectrum of the solution after each scan to ensure there was no sample degradation.

Because the spectral full-width at half-maximum of the mid-IR pulses was limited to at most ~175 cm^−1^, measurements were made piecewise from ~1300–1900 cm^−1^ for DOPA melanin solutions. The three regions measured were (1) 1290–1475 cm^−1^, (2) 1450–1700 cm^−1^, and (3) 1675–2000 cm^−1^. We took special care in properly calibrating the detected mid-IR frequency axis for each of these regions using the IR absorption lines of reference materials. For region 1, we used tetrahydrofuran, unstretched Parafilm, and a plastic film. For region 2, we used water vapor. For region 3, water vapor and trimethylamine were used.

For TRIR measurements on the Cat and Quin solutions, the on-axis excitation fluence was ~2 mJ cm^−2^ at all excitation wavelengths, assuming a Gaussian beam. Note that a broad Δ*A* signal, spanning the entire spectral window, is observed when exciting the dilute Cat+Quin mixture at 280 nm (Supplementary Fig. 8). This electronic background signal, which is observed when using UV pulses at high excitation fluence, was removed from the data as reported previously^52^, and as described in the Supplementary Note 6.

## Supplementary information

Supplementary Information

Peer Review File

## Data Availability

All experimental data presented in the main text and in Supplementary Information are available from the corresponding author upon request.
