# Supplementary material for: Probing the heterogeneous structure of eumelanin using ultrafast vibrational fingerprinting
Source: Nat Commun. 2020 Sep 11;11:4569. doi: 10.1038/s41467-020-18393-w (PMC7486937; doi:10.1038/s41467-020-18393-w)
Supplement: Supplementary file 1 — Supplementary Information [file 41467_2020_18393_MOESM1_ESM.pdf]

Supplementary Information

Probing the heterogeneous structure of eumelanin using ultrafast vibrational fingerprinting

Grieco et al.

Supplementary Note 1

The TRIR spectra of DOPA melanin at several delay times are compared after scaling for each of the excitation wavelengths tested in Supplementary Figure 1. The spectra agree within experimental uncertainty. The emergence of the hot D_2O signal over time is seen for all excitation wavelengths.

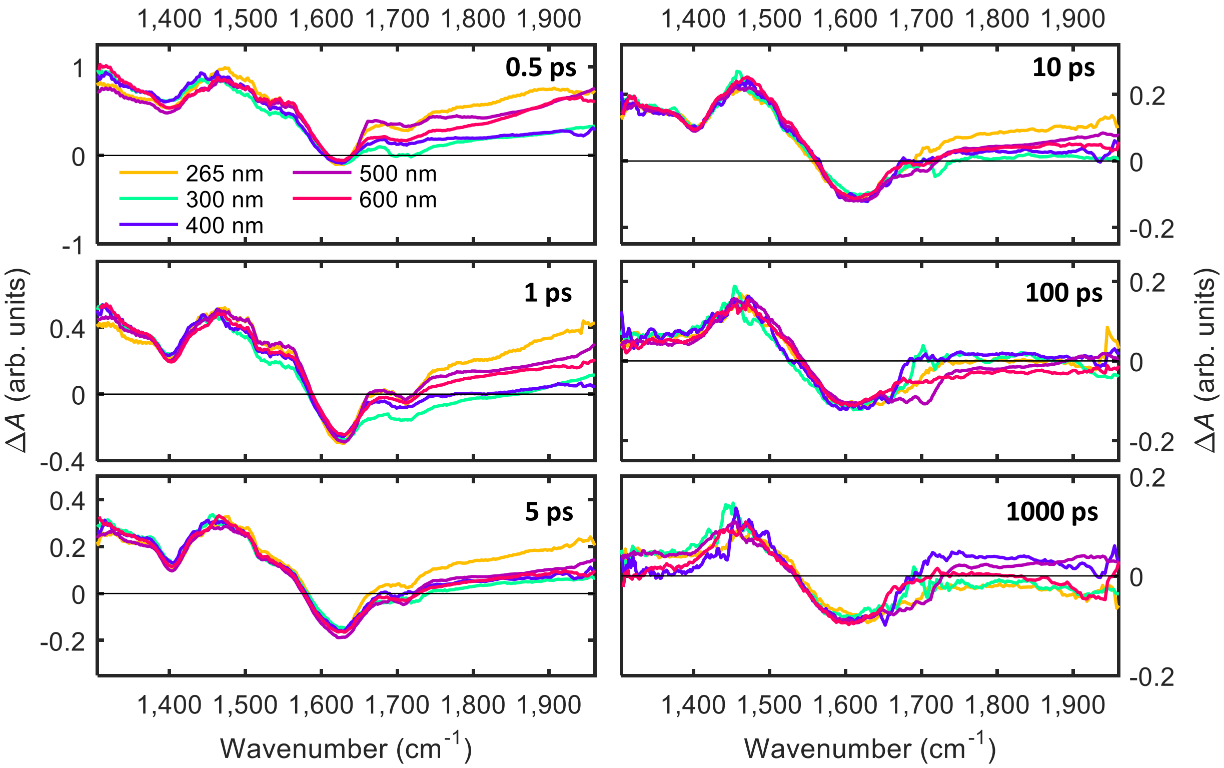

Supplementary Figure 1. Comparison of TRIR spectra of a DOPA melanin sample dissolved in phosphate buffered D_2O and excited using different UV and visible wavelengths, shown at the indicated delay times.

Supplementary Note 2

The time evolution of the TRIR spectrum of DOPA melanin is shown in Supplementary Figure 2 for each excitation wavelength studied except for 265 nm, which is included in the main text.

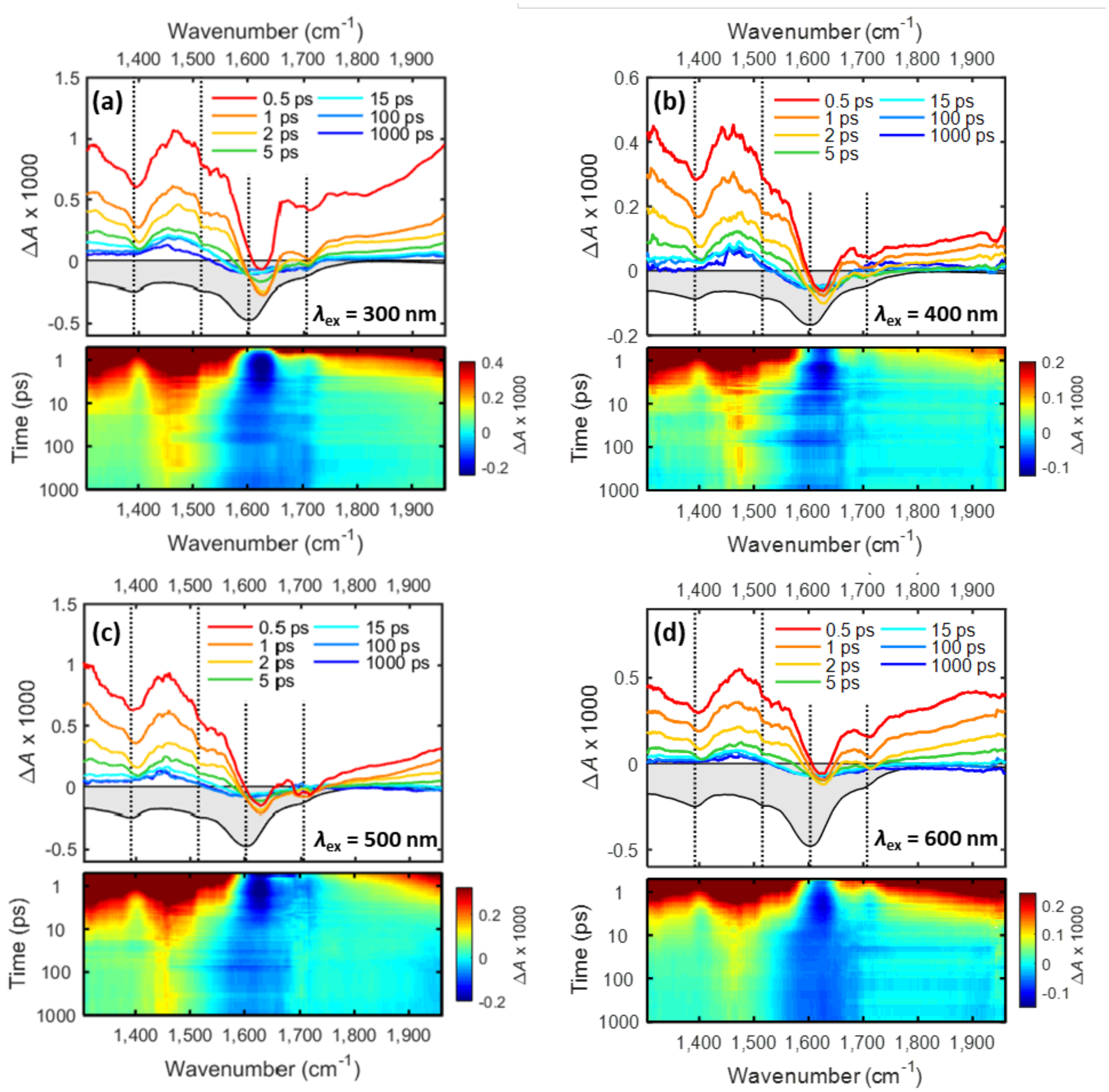

Supplementary Figure 2. Time evolution of the TRIR spectrum of DOPA melanin dissolved in phosphate-buffered D_2O, excited at (a) 300 nm, (b) 400 nm, (c) 500 nm, and (d) 600 nm. The inverted FTIR spectrum is shown in each graph in gray shading.

The TRIR kinetics averaged over 1750–1900 cm^{-1} together with fits to a stretched exponential decay function, $Ae^{-\left(\frac{t}{\tau}\right)^{\beta}}$, are shown in Supplementary Figure 3a for each excitation wavelength studied. The parameters of the stretched exponential function were fixed at the values reported previously for near-infrared transient absorption decays in DOPA melanin excited at 265 nm ($\tau = 0.114$ ps and $\beta = 0.33$),[1] while the amplitude, A, was freely optimized. Additionally, the kinetics at 1310 cm^{-1} from the main text are overlaid with a transient absorption decay recorded with 400 nm excitation and probed in the near-infrared in Supplementary Figure 3b.

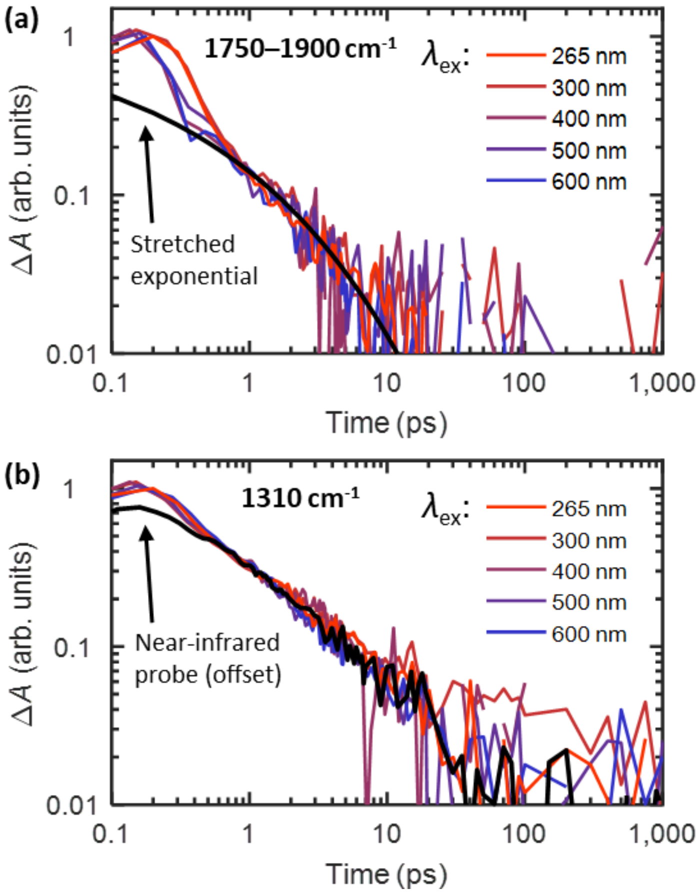

Supplementary Figure 3. (a) DOPA melanin TRIR kinetics averaged over 1750–1900 cm^{-1} for each excitation wavelength studied. The black trace is a stretched exponential function determined from a fit to the near infrared pump-probe signals from DOPA melanin with pump at 265 nm and probe at 950 nm from ref. 1. (b) DOPA melanin TRIR kinetics probed at 1310 cm^{-1} for each excitation wavelength studied. The black trace is a transient absorption decay measured using 400 nm excitation and probing at in the near infrared at 950 nm. The decay trace was negatively offset by 5% of the maximum intensity before scaling to highlight the similarity in the decays after 500 fs.

Supplementary Note 3

The differential FTIR spectrum of neat D_2O was measured as a function of temperature between 25°C and 65°C (Supplementary Figure 4). The IR intensity spectrum, $I(T)$, was recorded at each temperature, T, and the difference in absorption, $\Delta A(T)$, was calculated as

$$A(T) = -\log\left({}^{I(T)}\!/_{I(25^{\circ}\mathrm{C})}\right). \quad (1)$$

In this way, measuring a blank spectrum at each temperature is unnecessary, reducing the number of scans needed. It was assumed further that reflection losses of the CaF_2 windows of the liquid cell change negligibly over the temperature range studied.

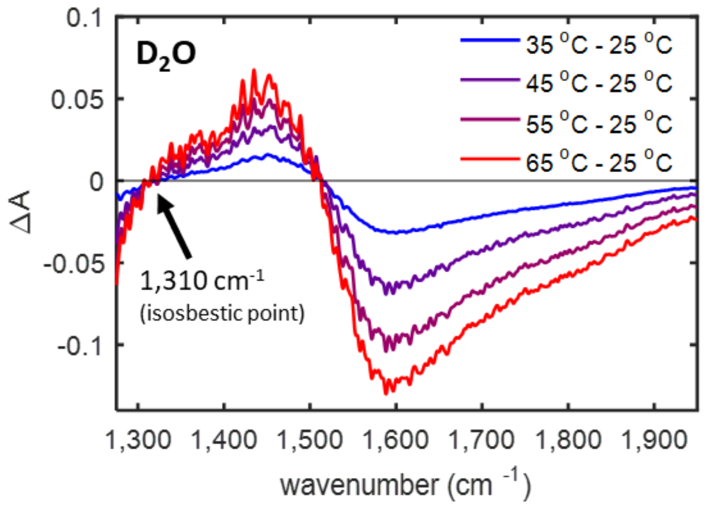

Supplementary Figure 4. Temperature difference FTIR spectra of D_2O relative to room temperature (25 °C). The D_2O sample was measured in a liquid cell with 1 mm and 2 mm thick CaF_2 windows and a path length of 100 µm.

Isosbestic points are detected when $\frac{\partial A}{\partial T} = 0$. Significantly, the difference spectra in Supplementary Figure 4 have zero intensity at 1310 cm^{-1}, indicating that the TRIR signals of DOPA melanin solutions do not contain contributions from hot D_2O at this frequency.

Supplementary Note 4

As indicated in the main text, the TRIR signals for DOPA melanin excited at 265 nm contain contributions from DOPA melanin and hot D_2O. In addition, a background signal arising from the CaF_2 windows and/or the solvent is also present (see Supplementary Discussion 5). The TRIR signal, $\Delta A(\tilde{\nu}, t)$, at time delay, t, and wavenumber, $\tilde{\nu}$, was modeled assuming additivity in the three signal contributions,

$$\Delta A(\tilde{\nu}, t) = \Delta A_{\mathrm{DOPA\ mel}}(\tilde{\nu}, t) + \Delta A_{\mathrm{Hot\ D_2O}}(\tilde{\nu}, t) + \Delta A_{\mathrm{bkgd}}(\tilde{\nu}, t), \quad (2)$$

arising from DOPA melanin excited states, $\Delta A_{\mathrm{DOPA\ mel}}$, hot D_2O molecules, $\Delta A_{\mathrm{Hot\ D_2O}}$, and a background signal, ΔA_{bkgd}. Each signal contribution is assumed to obey Beer's Law such that it increases linearly with the number density or concentration of the absorber. Furthermore, the signal from a constant number of absorbers is assumed to be invariant with time, i.e., absorption cross sections are time-independent. In this case, the signal at a time t' will increase or decrease compared to the signal at an earlier time t, depending solely on whether the number of absorbers increases or decreases, respectively. Each signal can be written as the product of the signal contribution from species X at time $t = t_{0,X}$, $\Delta A_X(\tilde{\nu}, t_{0,X}) \equiv \Delta A_X(\tilde{\nu})$, and a dimensionless population function, $c_X(t)$,

$$\Delta \mathrm{A_X}(\tilde{\nu}, t) = \Delta A_X(\tilde{\nu}) c_X(t). \quad (3)$$

The population function, which equals unity at $t = t_0$, describes the population kinetics of state X and is the quantity that we seek to determine by fitting. Writing 1 for "DOPA mel", 2 for "Hot D_2O", and 3 for the background signal, the total TRIR signal is given by,

$$\Delta A(\tilde{\nu}, t) = \Delta A_1(\tilde{\nu}) c_1(t) + \Delta A_2(\tilde{\nu}) c_2(t) + \Delta A_3(\tilde{\nu}) c_3(t), \quad (4)$$

The excellent agreement of the long-time signal with the FTIR temperature difference spectrum (blue and gray curves in Figure 5c of the main text) indicates that absorption by hot D_2O is the only significant signal contribution at delay times greater than 1 ns. Furthermore, experiments on a buffer-only solution described in Supplementary Note 5 show that the background signal has vanished by 100 ps after excitation (Supplementary Figure 7). The population functions, $c_1(t)$ and $c_3(t)$, thus both approach zero

at $t > 1$ ns because no signal from either DOPA melanin or the background are discernable then, so we use the signal averaged between 1 and 2 ns to estimate $\Delta A_2(\tilde{\nu})$,

$$\Delta A_2(\tilde{\nu}) = \Delta A(\tilde{\nu}, t > 1 \text{ ns}). \quad (5)$$

Next, the background signal contribution (index 3) at each delay time is assumed to have the same value at all frequencies consistent with the spectrally flat signals in Supplementary Figure 7. In this case, the background signal at a time, $t_{0,3}$, of our choosing is given by the constant ΔA_3,

$$\Delta A_3(\tilde{\nu}) = \Delta A_3 \quad (6)$$

Finally, the signal contribution from DOPA melanin is needed. This is more difficult to estimate from the total signal due to overlap with the other signal contributions. From equation 4, the signal at 0.5 ps is equal to,

$$\Delta A(\tilde{\nu}, t = 0.5 \text{ ps}) = \Delta A_1(\tilde{\nu}) c_1(t = 0.5 \text{ ps}) + \Delta A_2(\tilde{\nu}) c_2(t = 0.5 \text{ ps}) + \Delta A_3 \quad (7)$$

where in writing the last term of the previous equation, we have chosen $t_{0,3} = 0.5$ ps. Furthermore, if we assume that there has been negligible heating of the solvent at 0.5 ps, we can set $c_2(t = 0.5 \text{ ps}) \approx 0$ and re-arrange equation 7,

$$\Delta A_1(\tilde{\nu}) = \Delta A(\tilde{\nu}, t = 0.5 \text{ ps}) - \Delta A_3, \quad (8)$$

where use was made of $c_1(t_{0,1} = 0.5 \text{ ps}) = 1$. Combining equations 4 – 8, allows the signal to be expressed as,

$$\Delta A(\tilde{\nu}, t) = \Delta A(\tilde{\nu}, t = 0.5 \text{ ps}) c_1(t) + \Delta A(\tilde{\nu}, t > 1 \text{ ns}) c_2(t) + \gamma(t), \quad (9)$$

where $\gamma(t) = \Delta A_3[c_3(t) - c_1(t)]$. Equation 9 was used to fit the transient spectra at each delay time by linear least squares to obtain $c_1(t)$, $c_2(t)$, and $\gamma(t)$ given the basis spectra $\Delta A(\tilde{\nu}, t = 0.5 \text{ ps})$ and $\Delta A(\tilde{\nu}, t > 1 \text{ ns})$ (red and blue curves, respectively, in Figure 5c in the main text). As seen from equations 8 – 9, ΔA_3 is needed to solve for the signal contribution from DOPA melanin and to obtain the background signal kinetics, $c_3(t)$, but the kinetics of DOPA melanin and Hot D_2O are determined uniquely even without

this value. The results for $c_1(t)$ and $c_2(t)$ from least-squares fitting are shown by the red and blue curves, respectively, in Figure 5d in the main text.

To illustrate the fidelity of the fits, Supplementary Figure 5 shows several examples of the fitting procedure for several time delays for an arbitrarily selected value of $\Delta A_3 = 0.5 \times 10^{-3}$.

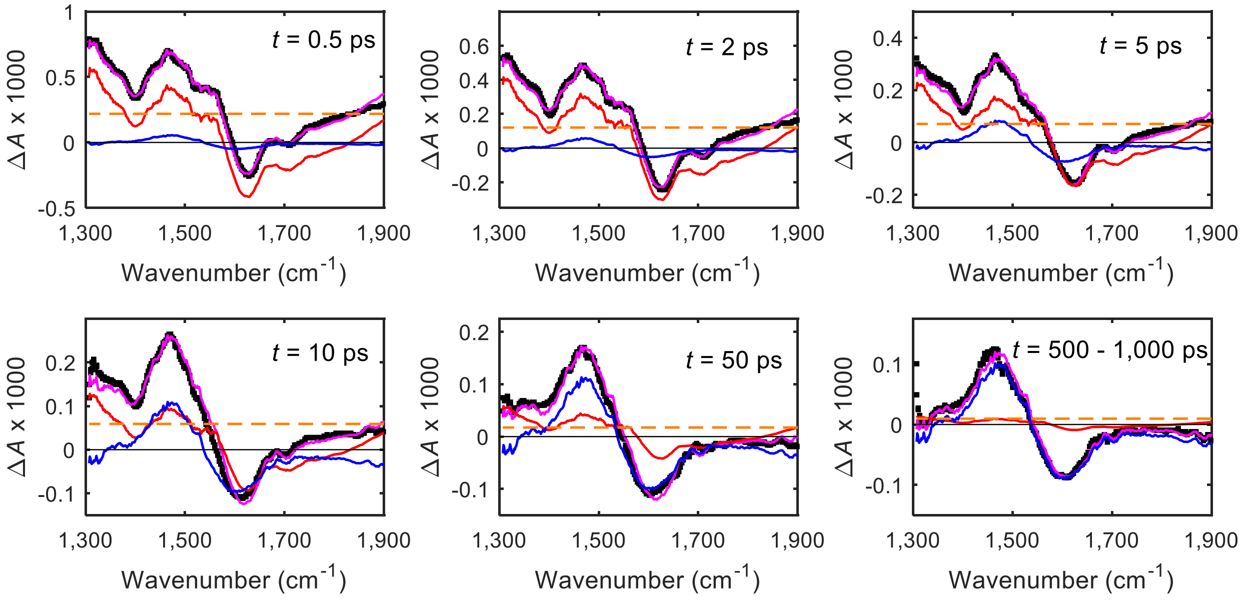

Supplementary Figure 5. Spectral decomposition of the TRIR spectra of DOPA melanin excited by 265 nm at several delay times. In each panel, the TRIR signal is show in black and the signal contributions from $\Delta A_{\mathrm{DOPA\ mel}}$, $\Delta A_{\mathrm{Hot\ D2O}}$, and ΔA_{bkgd} are shown in red, blue, and orange, respectively. The pink trace shows the sum of the red, blue, and orange curves.

Supplementary Figure 6 compares the DOPA melanin excited state population kinetics, $c_1(t)$, to the observed TRIR kinetics at 1310 cm^{-1}, a frequency, which has no contribution from the hot D_2O signal (Supplementary Figure 4). The excellent agreement of the two traces at $t > 0.5$ ps validates the fitting procedure described above. The kinetic decays in Supplementary Figure 6 can be described using a sum of a stretched

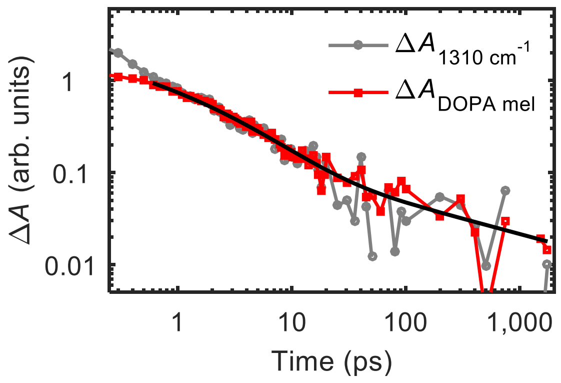

Supplementary Figure 6. Decomposed TRIR kinetics of the DOPA melanin signal compared to the observed kinetics probed at 1310 cm^{-1} for 265 nm excitation. The black trace is a fit to a sum of a stretched exponential and power law.

exponential and power law model, a model we tested with electronic transient absorption spectroscopy measurements probed in the near-IR region for an aqueous DOPA melanin solution.[1] Fixing the decay parameters to those we previously reported[1], but allowing the weights of the stretched exponential and power law to vary, produces the black trace in Supplementary Figure 6.

Supplementary Note 5

The high fluence UV pump pulses induce TRIR signals from the solvent and CaF_2 windows of the liquid cell. TRIR spectra of a phosphate buffered D_2O control sample were measured using 265 nm and 300 nm excitation (Supplementary Figure 7) under identical conditions (i.e., sample path length and excitation fluence) as measurements shown in the main text. The background spectrum appears as a broad positive offset and lacks vibrational features seen in the DOPA melanin sample measurements, indicating that the background signal is electronic in nature.

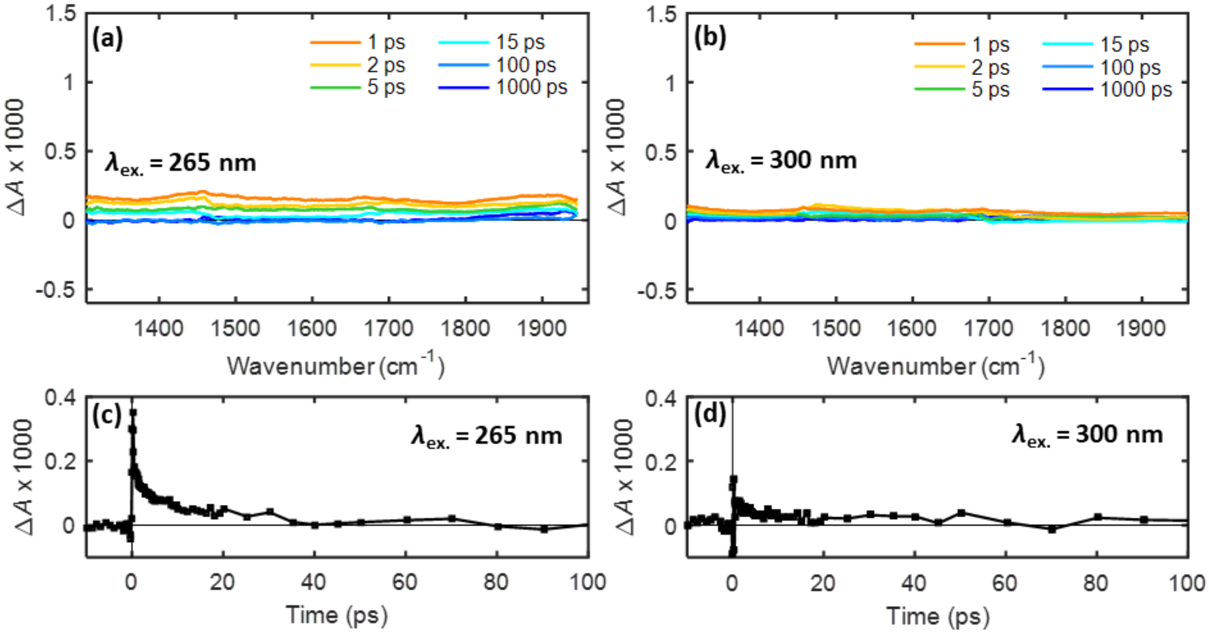

Supplementary Figure 7. Transient absorption spectra of phosphate buffered D_2O pumped at (a) 265 nm or (b) 300 nm with identical measurement conditions (i.e., pathlength and incident excitation energy density) used for DOPA melanin samples, shown on the same intensity scales. Decay kinetics obtained by averaging the transient absorption signal from ~1300 – 2000 cm^{-1} recorded using (c) 265 nm or (d) 300 nm.

Supplementary Note 6

A broad background signal in the TRIR spectrum is seen when exciting a dilute mixture of 3,5-di-*t*-butylcatechol (Cat) and 3,5-di-*t*-butyl-*o*-quinone (Quin) in cyclohexane with 265 nm light. Supplementary Figure 8a shows TRIR spectra with this signal present. The background signal was removed by subtracting a line that was fit to portions of the spectra lacking vibrational features at each time delay.[2] The results of this procedure are shown in Supplementary Figure 8b.

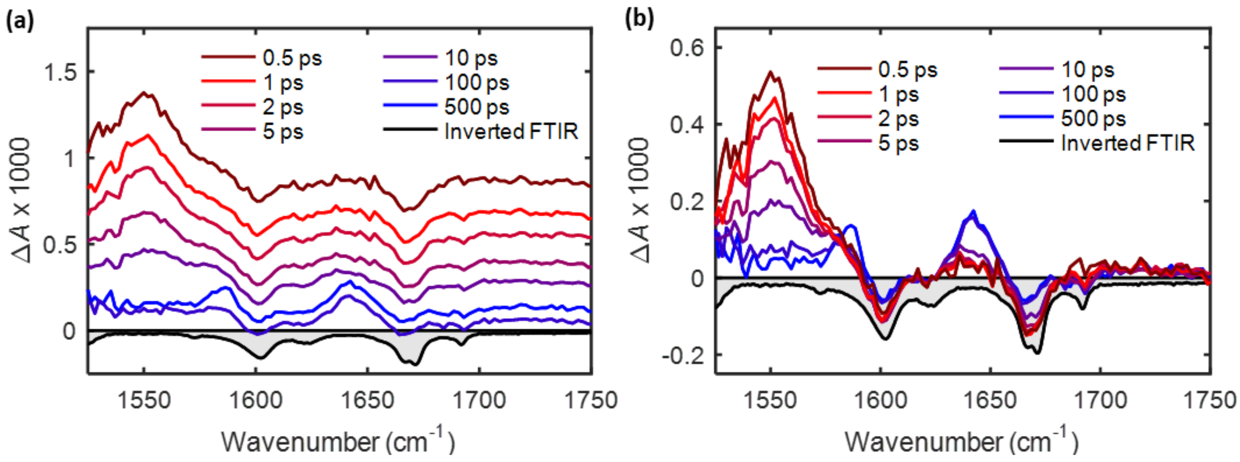

Supplementary Figure 8. TRIR spectra of a dilute Cat+Quin mixture recorded using 265 nm excitation (a) before and (b) after subtracting the broad electronic offset signal from the spectra at each time delay.

Supplementary References

1. Kohl, F. R., Grieco, C. & Kohler, B. Ultrafast spectral hole burning reveals the distinct chromophores in eumelanin and their common photoresponse. *Chem. Sci.* **11**, 1248-1259 (2020).

2. Grieco, C. *et al.* Intermolecular Hydrogen Bonding Modulates O-H Photodissociation in Molecular Aggregates of a Catechol Derivative. *Photochem Photobiol* **95**, 163-175 (2019).
