# Supplementary material for: Probing the heterogeneous structure of eumelanin using ultrafast vibrational fingerprinting
Source: Nat Commun. 2020 Sep 11;11:4569. doi: 10.1038/s41467-020-18393-w (PMC7486937; doi:10.1038/s41467-020-18393-w)
Supplement: Supplementary file 2 — Peer Review File [file 41467_2020_18393_MOESM2_ESM.pdf]

Editorial Note: This manuscript has been previously reviewed at another journal that is not operating a transparent peer review scheme. The manuscript was considered suitable for publication without further review at *Nature Communications.*
